# Impact of methodological "shortcuts" in conducting public health surveys: Results from a vaccination coverage survey

**DOI:** 10.1186/1471-2458-8-99

**Published:** 2008-03-27

**Authors:** Elizabeth T Luman, Mariana Sablan, Shannon Stokley, Mary M McCauley, Kate M Shaw

**Affiliations:** 1 National Center for Immunization and Respiratory Diseases, US Centers for Disease Control and Prevention, 1600 Clifton Rd, Atlanta GA 30333, USA; 2 Department of Health, Division of Public Health, PO Box 500409 CK, Saipan MP 96950v, Commonwealth of the Northern Mariana Islands

## Abstract

**Background:**

Lack of methodological rigor can cause survey error, leading to biased results and suboptimal public health response. This study focused on the potential impact of 3 methodological "shortcuts" pertaining to field surveys: relying on a single source for critical data, failing to repeatedly visit households to improve response rates, and excluding remote areas.

**Methods:**

In a vaccination coverage survey of young children conducted in the Commonwealth of the Northern Mariana Islands in July 2005, 3 sources of vaccination information were used, multiple follow-up visits were made, and all inhabited areas were included in the sampling frame. Results are calculated with and without these strategies.

**Results:**

Most children had at least 2 sources of data; vaccination coverage estimated from any single source was substantially lower than from all sources combined. Eligibility was ascertained for 79% of households after the initial visit and for 94% of households after follow-up visits; vaccination coverage rates were similar with and without follow-up. Coverage among children on remote islands differed substantially from that of their counterparts on the main island indicating a programmatic need for locality-specific information; excluding remote islands from the survey would have had little effect on overall estimates due to small populations and divergent results.

**Conclusion:**

Strategies to reduce sources of survey error should be maximized in public health surveys. The impact of the 3 strategies illustrated here will vary depending on the primary outcomes of interest and local situations. Survey limitations such as potential for error should be well-documented, and the likely direction and magnitude of bias should be considered.

## Background

In large state-of-the art public health surveys, such as UNICEF's Multiple Indicator Cluster Surveys [1], USAID's Demographic and Health Surveys [2], and the US Centers for Disease Control and Prevention's (CDC) National Immunization Survey [3] and National Health Interview Survey [4], much effort and expense are dedicated to maximizing representativeness and validity of results. This is done by incorporating strategies to reduce the impact of various sources of error, such as sampling error, measurement error, nonresponse error, and noncoverage error. However, in smaller field surveys worldwide, limited time and financial resources, poor accessibility of households, and lack of available transportation and staff may limit the extent to which these strategies can be implemented. While these surveys often provide practical information used as management tools for evaluating and targeting health services, in extreme cases survey error could result in severe bias, proliferation of misinformation, and suboptimal public health response.

Previously published research and discussion have focused on sampling error and the sampling methods used in field surveys [5-12]; however, several other issues are frequently overlooked. In this study we examine additional sources of survey error and the potential impact of three "shortcuts" that are sometimes taken when conducting household surveys:

• Use of only a single or inaccurate source of information for critical outcomes, which may cause measurement error

• Not revisiting households that are unavailable for interview at the initial visit, which can contribute to nonresponse error, and

• Excluding areas that are difficult to access or are far from primary population centers, which can result in noncoverage error.

To demonstrate these points, we used data from a vaccination coverage survey of young children conducted in the US Commonwealth of the Northern Mariana Islands (CNMI) in July 2005. In this survey, three sources of vaccination history information were used, multiple follow-up attempts were made to interview household members, and all inhabited areas were included in the sampling frame. We examine the impact of these efforts and when possible, estimate the added time that was required to incorporate each of them into the survey. We also illustrate how a simple sensitivity analysis can be used to estimate potential bias in surveys that exclude portions of a population. Finally, we discuss the factors that might influence the magnitude of the impact of each of these "shortcuts" in various settings.

All surveys are subject to sources of error [13], and understanding how error may bias survey results is crucial for drawing appropriate conclusions and response. The objectives of this report are to increase awareness of potential sources of survey error, to encourage survey researchers to implement strategies that minimize these sources of error, and to remind data users of the need to critically assess the extent to which survey results may be biased.

## Methods

### Setting and survey description

CNMI is located in the Western Pacific Ocean between Australia and Japan, and is composed of a chain of 15 islands that extends 400 nautical miles. The population of CNMI is approximately 70,000 people, with an annual birth cohort of approximately 1300 children on its three inhabited islands (1,130 on Saipan, 85 on each of Rota and Tinian) [14]. Rota and Tinian, located 73 and five miles from Saipan, respectively, are accessible via daily commuter flights from Saipan. Due to their relatively small populations and remoteness, many health services are provided to residents of these islands through periodic outreach activities.

The primary objective of this survey was to estimate the percentages of children aged one, two, and six years who had received the standard vaccination series recommended for their ages. Among children aged one year (12–23 months), we evaluated receipt of vaccines recommended by age 12 months: three doses of diphtheria-pertussis-tetanus vaccine (DPT), three doses of inactivated poliovirus vaccine (IPV), two doses of hepatitis B vaccine, and three doses of *Haemophilus influenzae *type b vaccine (Hib). Among children aged two years (24–35 months) we evaluated receipt of vaccines recommended by age 24 months: four doses of DPT, three doses of IPV, one dose of measles-mumps-rubella vaccine (MMR), three doses of hepatitis B vaccine, and three doses of Hib. For children aged six years, we evaluated receipt of vaccine doses required for school entry: five doses DPT, four doses of IPV, two doses of MMR, and three doses of hepatitis B vaccine.

On Saipan, the largest of the three islands, a population-based cluster survey was conducted. District population estimates were obtained from the most recent census, conducted in 2000. Thirty clusters were selected by systematic random sampling with probability of selection proportional to estimated size of district populations. Next, households were chosen by systematic random sampling in the selected clusters. A household was defined as a group of persons who live and eat together; children who usually resided in one household but were temporarily staying in another were included in the household where they usually resided. A household was considered eligible for the survey if there was at least one child aged one, two, or six years currently residing there. A target sample size of 22 children per age group per cluster was set so that estimated vaccination coverage would be within 7% of true coverage with α = .05, assuming a design effect of two and an expected response rate of 90%. The sampling interval (*i*) was determined for each cluster by dividing the estimated number of age-eligible children by the target sample size. Interviewers proceeded in a serpentine manner throughout all inhabited areas of each selected cluster and visited every *i*th household to determine eligibility. All eligible children in selected households were included. Interviewers continued within a cluster until the entire cluster was canvassed, regardless of the number of households visited or children surveyed. On Rota and Tinian every household was visited due to small population size.

Interviews were conducted by nine teams of two health workers. Before conducting the interview, interviewers explained the purpose of the study and the respondent was given the opportunity to ask questions about the survey. Written informed consent from a parent or legal guardian was requested for participation in the household survey and to obtain vaccination records from the electronic registry and the health department. This study was approved by CDC's institutional review board, and additional details have been reported elsewhere [15].

### Using multiple sources of vaccination history information

Vaccination histories were obtained from three separate sources of information. First, household-retained vaccination cards were reviewed and transcribed during the household interviews. Second, health records were obtained from the public health department for study children by matching the child's name, date of birth, and hospital identification number; vaccination histories were abstracted from these records. Third, vaccination histories for study children were also obtained from the computerized vaccination registry, which was implemented in 1989. In cases where the vaccination cards, health records, and vaccination registry were not identical, vaccinations recorded in the three sources were combined. We evaluated vaccination coverage based on each source of data independently, as well as vaccination coverage when all sources of information were combined. We also examined agreement of sources, assessing the number of independent sources that reported each child as completely vaccinated.

### Revisiting households not available at the initial visit

If no adult respondent was home at the time of the initial visit, interviewers returned to the household on evenings or weekends to interview the household. At least two such follow-up visits were made for each household. We evaluated eligibility ascertainment rates, eligibility rates, and vaccination coverage rates that would have been achieved if the study had been conducted with and without these follow-up visits.

### Including areas that are difficult to access

We estimated vaccination coverage rates for each of the three inhabited islands of CNMI. Rota and Tinian are difficult to access due to their remoteness from the main island of Saipan. To determine the effect of including these difficult-to-access areas, we compared vaccination coverage based on results from Saipan alone with results obtained by estimating vaccination coverage as a weighted average of all three islands.

### Statistical analysis

Data entry and cleaning were conducted in Epi Info 2002 version 3.3.2 (Centers for Disease Control and Prevention, GA) and SAS version 8.0 (SAS Institute, NC). Analyses were conducted in SUDAAN version 9.0.0 (Research Triangle Institute, NC).

Bivariate analyses were used to estimate vaccination coverage and 95% confidence intervals for children identified with and without follow-up, for each island and for CNMI overall, and by source of vaccination information. All analyses account for the sampling design and were weighted to adjust for differences in probability of selection.

In a survey with excluded populations, a simple sensitivity analysis can be conducted to determine the likely magnitude of potential survey bias. We conducted a sensitivity analysis of potential bias due to exclusion of children in households for which eligibility was not determined. We assumed that children in households with unknown eligibility were twice as likely to be unvaccinated (lower bound) and half as likely to be unvaccinated (upper bound) as those included. By combining these estimates with those for children who were included, we calculated lower and upper bounds of the likely results accounting for the potential bias. For illustrative purposes, we conducted similar sensitivity analyses based on children accessed at initial visits to households, and those living on the main island of Saipan. We then compared these ranges with the observed coverage for the entire sample.

## Results

### Using multiple sources of vaccination history information

Overall, vaccination history information was available from household-retained vaccination cards for 71%, 70%, and 59% of children aged one, two, and six years, respectively (Table 1). Vaccination histories were obtained from health department records for 87%, 88%, and 91%, respectively, and from the electronic vaccination registry for 98%, 96%, and 70%, respectively. Most children had at least two sources of data (95%, 97%, and 87%, respectively).

**Table 1:** Percent of children with each source of information and number of sources. Commonwealth of the Northern Mariana Islands, 2005.

	Source						Number of Sources							
		
	Vaccination Card		Health Department Record Abstraction		Vaccination Registry		3 Sources		2 Sources		1 Source		0 Sources	
Age (y)	%	95% CI	%	95% CI	%	95% CI	%	95% CI	%	95% CI	%	95% CI	%	95% CI
1	70.9	65.0, 76.8	87.4	83.9, 90.9	98.2	96.6, 99.8	61.8	55.7, 67.9	33.1	27.2, 39.0	5.0	2.5, 7.5	0.2	0.0, 0.4
2	70.4	64.1, 76.7	88.2	84.3, 92.1	96.4	93.9, 98.9	59.3	52.6, 66.0	37.2	30.6, 43.8	2.6	1.1, 4.1	0.9	0.0, 2.2
6	58.5	51.8, 65.2	91.4	88.3, 94.5	69.7	63.4, 76.0	33.2	26.6, 39.8	53.5	46.5, 60.5	13.0	8.6, 17.4	0.3	0.2, 0.4

Estimated vaccination coverage calculated from any single source was substantially lower than from all sources combined (Table 2). Coverage estimated by vaccination cards was lowest: 38%, 32%, and 22% among children aged one, two, and six years, respectively. Coverage estimated by abstraction of health department records was somewhat higher: 60%, 34%, and 49%, respectively. Vaccination registries generally produced the highest vaccination coverage rates: 85%, 70%, and 48%, respectively. With all sources combined, coverage rates were 92%, 82%, and 83%, respectively.

**Table 2:** Estimated vaccination coverage* based on source of information^†^. Commonwealth of the Northern Mariana Islands, 2005.

	Vaccination Card		Health Department Record Abstraction		Vaccination Registry		All Sources Combined	
				
	%	95% CI	%	95% CI	%	95% CI	%	95% CI
1 year	37.5	31.2, 43.8	59.8	53.5, 66.1	84.6	80.5, 88.7	91.8	88.3, 95.3
2 years	32.0	25.3, 38.7	34.3	28.0, 40.6	69.6	63.3, 75.9	82.4	77.3, 87.5
6 years	21.6	15.9, 27.3	49.1	42.2, 56.0	47.7	40.8, 54.6	83.0	77.9, 88.1

*Children aged 1 year: received at least 3 doses of diphtheria-pertussis-tetanus vaccine (DPT), 3 doses of inactivated poliovirus vaccine (IPV), 2 doses of *Haemophilus influenzae *type b vaccine (Hib) and 3 doses of hepatitis B vaccine. Children aged 2 years: received at least 4 doses of DPT, 3 doses of IPV, 1 dose of measles-mumps-rubella vaccine (MMR), 3 doses of Hib, and 3 doses of hepatitis B. Children aged 6 years: received at least 5 doses DPT, 4 doses of IPV, 2 doses of MMR, and 3 doses of hepatitis B vaccine.

^†^Children without information are considered not vaccinated.

Many children were found to be completely vaccinated in two or more independent sources (Table 3). Among one-year-old children, 23% were reported as completely vaccinated in each of the three sources independently, 46% in two of the three sources (most commonly the health record and vaccination registry), and 21% in only one source (most commonly the vaccination registry). Approximately 2% of one-year-old children did not have evidence of complete vaccination in any single source, but were determined fully vaccinated only after combining vaccinations documented in multiple sources. Among two-year-old children, 11% were reported completely vaccinated in each of the three sources, 37% in two of the three sources, and 30% in only one source; 5% required combining records to be considered completely vaccinated. Among six-year-old children, fewer were reported completely vaccinated in three or two sources (9% and 28%, respectively), and more were completely vaccinated in only one source (37%) or after combining sources (9%).

**Table 3:** Agreement of sources: percentage of children reported completely vaccinated* in 1, 2, 3, and 0 sources. Commonwealth of the Northern Mariana Islands, 2005.

Number of sources independently reporting child completely vaccinated	1 year			2 years			6 years		
			
	n	%	95%CI	n	%	95%CI	n	%	95%CI
**1 Source**	**58**	**20.7**	**15.8, 25.6**	**54**	**29.6**	**23.1, 36.1**	**74**	**37.3**	**30.6, 44.0**
Vaccination Card	7	1.1	0.8, 1.4	7	2.8	0.8, 4.8	9	4.6	1.7, 7.5
Health Department Record Abstraction	13	2.7	1.3, 4.1	8	3.5	1.1, 5.9	32	16.0	11.1, 20.9
Vaccination Registry	38	16.9	12.2,21.6	39	23.4	17.3, 29.5	33	16.7	11.6, 21.8
									
**2 Sources**	**97**	**45.7**	**39.2, 52.2**	**71**	**37.0**	**30.3, 43.7**	**53**	**27.7**	**21.4, 34.0**
Vaccination Card and Health Department Record Abstraction	6	1.4	0.2, 2.6	3	1.6	0.0, 3.2	9	5.2	1.9, 8.5
Vaccination Card and Vaccination Registry	26	11.8	7.5, 16.1	25	17.0	11.3, 22.7	7	3.2	0.8, 5.6
Health Department Record Abstraction and Vaccination Registry	65	32.5	26.4, 38.6	43	18.5	13.4, 23.6	37	19.2	13.7, 24.7
									
**All 3 Sources**	**45**	**23.2**	**17.7, 28.7**	**23**	**10.7**	**6.6, 14.8**	**15**	**8.6**	**4.7, 12.5**
									
**0 Sources**	**24**	**10.3**	**6.6, 14.0**	**46**	**22.7**	**17.0,28.4**	**51**	**26.5**	**20.4, 32.6**
Completely vaccinated only when information from multiple sources was combined	5	2.1	0.3, 3.9	11	5.0	2.1, 7.9	17	9.4	5.5, 13.3
Not Completely Vaccinated	19	8.3	4.8, 11.8	35	17.6	12.5, 22.7	34	17.0	11.9, 22.1

*Children aged 1 year: received at least 3 doses of diphtheria-pertussis-tetanus vaccine (DPT), 3 doses of inactivated poliovirus vaccine (IPV), 2 doses of *Haemophilus influenzae *type b vaccine (Hib) and 3 doses of hepatitis B vaccine. Children aged 2 years: received at least 4 doses of DPT, 3 doses of IPV, 1 dose of measles-mumps-rubella vaccine (MMR), 3 doses of Hib, and 3 doses of hepatitis B. Children aged 6 years: received at least 5 doses DPT, 4 doses of IPV, 2 doses of MMR, and 3 doses of hepatitis B vaccine.

Abstraction of health records was conducted by two people on Saipan and one person each on Rota and Tinian. An estimated 16 person-hours were required for data abstraction, or 6% of the total person-hours for the survey (Table 4). Because the vaccination registry was already computerized, accessing its data did not require any additional survey time.

**Table 4:** Estimated person-days required to complete selected portions* of a vaccination coverage survey. Commonwealth of the Northern Mariana Islands, 2005.

	Estimated Person-Days							Number of	
		
	Travel^†^	Training	Supervision	Interviews	Health Department Record Abstraction	Data Entry	Total	Households Visited	Child Interviews
Saipan	0	30	30	100	14	20	194	2870	414
Rota	3	4	1	16	1	3	28	558	76
Tinian	3	4	1	16	1	2	27	680	121
Total	6	38	32	132	16	25	249	4108	611

*Excludes pre- and post- survey activities: survey development and preparation, logistical planning, data cleaning and analysis, and writing of final report.

^†^Interviewers from Rota and Tinian traveled to Saipan for training.

### Revisiting households not available at the initial visit

**Figure 1:**
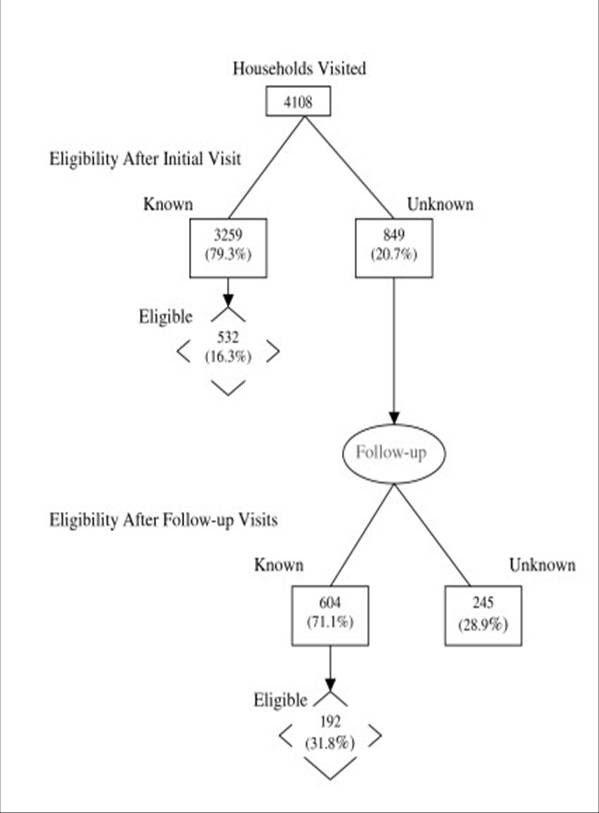
Sample sizes and eligibility. Households with one or more children aged one, two, or six years were eligible for the study.

After the initial visit, eligibility was known for 79% of households and 16% of them had one or more age-eligible children (Figure 1). Among the households with unknown eligibility after the initial visit, interviewers were able to determine eligibility for 71% after follow-up visits, yielding an additional 192 eligible households. After follow-up, eligibility was known for 94% of households; 19% of them were eligible and <1% of eligible households refused to participate.

Vaccination coverage rates among children in all households were similar to those among their counterparts in households available at the initial visit (Table 5). Confidence intervals were narrower when the survey was conducted with follow-up visits (7%–10% compared to 8%–13%) due to the increased sample size obtained.

**Table 5:** Effect of conducting follow-up for households not available at initial visit. Commonwealth of the Northern Mariana Islands, 2005.

	Study Conducted	
	
	Without Follow-up	With Follow-up
Number of households with known eligibility	3259	3863
Percentage of households with known eligibility	79.3%	94.0%
Number of eligible households	532	724
Percentage of households eligible	16.3%	18.7%
Number of children		
1 year	175	224
2 years	145	194
6 years	151	193
Estimated % of children vaccinated* (95% CI)		
1 year	91.5% (87.6, 95.4)	91.8% (88.3, 95.3)
2 years	80.2% (73.9, 86.5)	82.4% (77.3, 87.5)
6 years	83.3% (77.6, 89.0)	83.0% (77.9, 88.1)

*Children aged 1 year: received at least 3 doses of diphtheria-pertussis-tetanus vaccine (DPT), 3 doses of inactivated poliovirus vaccine (IPV), 2 doses of *Haemophilus influenzae *type b vaccine (Hib) and 3 doses of hepatitis B vaccine. Children aged 2 years: received at least 4 doses of DPT, 3 doses of IPV, 1 dose of measles-mumps-rubella vaccine (MMR), 3 doses of Hib, and 3 doses of hepatitis B. Children aged 6 years: received at least 5 doses DPT, 4 doses of IPV, 2 doses of MMR, and 3 doses of hepatitis B vaccine.

### Including areas that are difficult to access

Vaccination coverage rates varied considerably by island (Table 6), with highest rates among children on Tinian and lowest rates among those on Rota. Estimated coverage rates for Saipan alone were within one percentage point of those for CNMI overall.

**Table 6:** Estimated vaccination coverage* among children living on the 3 inhabited islands of the Commonwealth of the Northern Mariana Islands, 2005.

	**Saipan**		**Rota**		**Tinian**		**CNMI**	
				
	**% Vaccinated**	**95% CI**	**% Vaccinated**	**95% CI**	**% Vaccinated**	**95% CI**	**% Vaccinated**	**95% CI**
1 year	91.9%	88.0, 95.8	82.4%	77,5, 87.3	97.6%	96.0, 99.2	91.8%	88.3, 95.3
2 years	82.1%	76.4, 87.8	65.0%	56.4, 73.6	93.2%	91.2, 95.2	82.4%	77.3, 87.5
6 years	83.2%	77.5, 88.9	59.1%	54.0, 64.2	94.4%	94.0, 94.8	83.0%	77.9, 88.1

	**N**	**%**	**N**	**%**	**N**	**%**	**N**	

Estimated Population^†^	3019	88	211	6	185	6	3415	

*Children aged 1 year: received at least 3 doses of diphtheria-pertussis-tetanus vaccine (DPT), 3 doses of inactivated poliovirus vaccine (IPV), 2 doses of *Haemophilus influenzae *type b vaccine (Hib) and 3 doses of hepatitis B vaccine. Children aged 2 years: received at least 4 doses of DPT, 3 doses of IPV, 1 dose of measles-mumps-rubella vaccine (MMR), 3 doses of Hib, and 3 doses of hepatitis B. Children aged 6 years: received at least 5 doses DPT, 4 doses of IPV, 2 doses of MMR, and 3 doses of hepatitis B vaccine.

^†^Number of children aged 1, 2, and 6 years, 2000 Census.

Note: Chi-squared test for differences between islands: factor-level p-value < .0001 for each age group.

Of the estimated 249 person-hours required to complete the survey, approximately 78% were spent to conduct the survey on Saipan, with 11% each on Rota and Tinian (Table 4).

### Sensitivity analysis

In the current study, eligibility was not ascertained for 245 of the 4,108 households visited (6%). If children living in these households were twice as likely to be unvaccinated as those in households for which eligibility was determined, their estimated coverage would be 88%, 74%, and 75% for children aged one, two, and six years, respectively (Table 7). If they were half as likely to be unvaccinated, their coverage would be 96%, 91%, and 92%, respectively. These estimates form a range of potential coverage that could be assumed for the excluded children. Calculating a weighted average of these estimates along with estimates for children in households for which eligibility was determined yields coverage ranging from 91.6%–92%, 82%–83%, and 83%–84% for children aged one, two, and six years, respectively. These are the likely ranges of overall coverage taking into account the potential bias of excluding children in households for which eligibility was not determined.

**Table 7:** Sensitivity analysis: potential bias introduced by excluding a proportion of the population. Commonwealth of the Northern Mariana Islands, 2005.

			Estimated Vaccination Coverage*		
			
	% of Population		1 year	2 years	6 years
Eligibility Determined in Study					
Determined	94.0		91.8	82.4	83.0
Not Determined^†^	6.0	Lower Bound	87.7	73.6	74.5
		Upper Bound	95.9	91.2	91.5
Weighted Average		Lower Bound	91.6	81.9	82.5
		Upper Bound	92.0	82.9	83.5
Availability at Initial Visit					
Available	79.3		91.5	80.2	83.3
Not Available^‡^	20.7	Lower Bound	83.0	60.4	66.6
		Upper Bound	95.8	90.1	91.7
Weighted Average		Lower Bound	89.7	76.1	79.8
		Upper Bound	92.4	82.2	85.0
Accessibility					
Saipan	88.4		91.9	82.1	83.2
Rota/Tinian^§^	11.6	Lower Bound	83.8	64.2	66.4
		Upper Bound	96.0	91.1	91.6
Weighted Average		Lower Bound	91.0	80.0	81.3
		Upper Bound	92.4	83.1	84.2
Observed Coverage			91.8	82.4	83.0

*Children aged 1 year: received at least 3 doses of diphtheria-pertussis-tetanus vaccine (DPT), 3 doses of inactivated poliovirus vaccine (IPV), 2 doses of *Haemophilus influenzae *type b vaccine (Hib) and 3 doses of hepatitis B vaccine. Children aged 2 years: received at least 4 doses of DPT, 3 doses of IPV, 1 dose of measles-mumps-rubella vaccine (MMR), 3 doses of Hib, and 3 doses of hepatitis B. Children aged 6 years: received at least 5 doses DPT, 4 doses of IPV, 2 doses of MMR, and 3 doses of hepatitis B vaccine.

^†^Coverage assuming children in households for which eligibility was not determined were twice as likely to be unvaccinated (lower bound) and half as likely to be unvaccinated (upper bound) as those for which eligibility was determined in the study.

^‡^Coverage assuming children not available were twice as likely to be unvaccinated (lower bound) and half as likely to be unvaccinated (upper bound) as those available at the initial visit.

^§^Coverage assuming children living on Rota and Tinian were twice as likely to be unvaccinated (lower bound) and half as likely to be unvaccinated (upper bound) as those on Saipan.

If we had not returned to households that were unavailable at the initial visit, a substantial proportion of children (21%) would have been excluded from the survey (Figure 1). If we were to assume that children in unavailable households were half to twice as likely to be unvaccinated as those in households that were available at the initial visit, their coverage would range from 83%–96% for children aged one year, 60%–90% for children aged two years, and 67%–92% for children aged six years. A weighted average taking into account the hypothetical bias of excluding children in households not available at the initial visit would yield coverage ranging from 90%–92%, 76%–82%, and 80%–85% for children aged one, two, and six years, respectively.

Similarly, if Rota and Tinian had been excluded from the sampling frame, we might assume that children living there were half to twice as likely to be unvaccinated as those living on Saipan. This assumption would yield likely ranges for overall coverage of 91%–92%, 80%–83%, and 81%–84% for children aged one, two, and six years, respectively.

## Discussion

Public health surveys are widely used by national, state, and local health departments as a basis for programmatic and policy decisions – to evaluate services, to target additional services, and to assess the success of these services in improving the health of the population. To be effective, surveys must produce reliable results that can be generalized to the population of interest. Ensuring methodological rigor is critical to reduce the potential for bias and thus provide a complete and accurate picture of the current situation, reveal strengths and weaknesses of health programs, and enable data-driven solutions. Several factors must be carefully considered when designing a public health survey: 1) the goals and intended uses of the study, 2) statistical properties of the methods, and 3) the relative feasibility of the options, given time and resource constraints, technical expertise, and geographic conditions. A successful survey must balance these factors to ensure that feasibility is maximized while methods are rigorous enough to produce results that are sufficiently precise and free of bias to be used for key program and policy decisions. A poorly conducted survey can produce erroneous results and may prompt inappropriate public health action. Overestimating the health problem or misidentifying risk groups can lead to limited resources being wasted that could be used more efficiently elsewhere, while underestimating the problem can lead to a false sense of security and lack of action needed to protect the population. Equally problematic, findings from a poorly conducted survey will be difficult to defend and may be disregarded, resulting in lack of political will to take action to correct the identified problems.

In this study, use of multiple sources of data had a substantial impact on the results. The discrepancy of results based on the different sources of data is striking, and emphasizes the importance of identifying reliable sources of information. Furthermore, estimates that would have been obtained with any one of the three sources would have been substantially lower than those achieved by combining the sources, and would have called for a different public health response. Completeness and accuracy of data are critical to the validity of any survey, whether the data represent respondent opinions, written records, physical measurements, or laboratory tests. While medical records may be the most accurate source for vaccination history information, they were nevertheless incomplete in this study. Furthermore, household surveys are conducted to ensure a population-based sample, and thus avoid inherent biases of sampling from medical records. Therefore, most vaccination coverage surveys rely on household-retained vaccination cards to obtain vaccination histories. While approximately 70% of one and two-year-old children in CNMI had vaccination cards, vaccination coverage based on these cards alone was less than half of that obtained by combining cards with the two other sources of information. Abstracting vaccination information from health department records increased total person-time to complete the survey by 6%; adding data from the electronic registry did not increase survey time.

We found that conducting follow-up visits to households not available at the initial visit did not substantially affect the outcome of interest; however, it improved our ability to determine household eligibility and substantially increased response rates. In addition, households whose eligibility was determined after follow-up were twice as likely to be eligible as those determined at the initial visit. It may not be possible to infer characteristics of the nonrespondents; high response rates help to minimize the size of this population and thus ensure the representativeness of those included in the survey. As a result, high response rates confer greater credibility and generalizability of the survey results. In addition to revisiting households, response rates can often be improved by promoting awareness of the survey, using well-designed questionnaires that minimize respondent burden, and providing adequate training to interviewers regarding the survey goals and interviewing techniques.

Excluding populations that are difficult to access is often tempting, due to high travel cost and time, as well as safety concerns in some settings. However, excluding portions of the population can lead to biased results if the characteristics of the excluded population differ from those included. In the United States, the federal Office of Management and Budget (OMB) has developed standards and guidelines for statistical surveys conducted by government agencies, recommending that the sampling frame cover at least 95% of the target population [16]. In the current study, exclusion of the two remote islands would have yielded survey coverage of 88% of the population, increasing the likelihood for biased results. Despite the opportunity for bias, excluding these islands actually had little effect on the overall estimates for CNMI, due in part to the divergent results on the two remote islands. Nevertheless, including these difficult-to-access areas provided important information for public health response: outreach activities appear to be working well on Tinian, while substantial problems were revealed that need to be addressed on Rota. Including Rota and Tinian increased the total person-time to complete the survey by 29%.

The effects of each of the three "shortcuts" evaluated in this study may vary depending on the primary outcomes of interest, population subgroup, and local situations. For example, in a setting with more complete health history documentation, or in a survey relying on physical measurements or laboratory tests, a single source of information may be sufficient. Estimating the effect of incomplete information or measurement error can be difficult. In surveys limited to one source of data, effort should be made to investigate and describe the level of accuracy and completeness of that source through objective measures and expert opinion.

The potential effect of excluding a portion of the population, either due to nonresponse or noncoverage, could be substantial, depending on the size of the population excluded relative to that included, and the difference in outcome characteristics between those included and those excluded. Thus, while low response rates and low coverage rates do not necessarily result in bias, they are an important indicator of the potential for bias. The US OMB suggests that if >15% of the population is excluded, or if overall response rates are <80%, a noncoverage or nonresponse bias analysis should be conducted [16]. Ideally, this would involve observing or measuring some characteristics of nonrespondents to better model their likely survey responses. However, if such information is not available, a simple sensitivity analysis, such as presented here, can be conducted to determine the limits of the bias introduced. Assuming that the likelihood of nonvaccination among children who would have been excluded from the survey was half to twice as much as for those included yielded ranges that, for the most part, contained the actual observed coverage estimates. However, assumptions regarding the excluded population should be made with care. For example, if outreach services are believed to be poor, lower bounds could be established that assume that none of the difficult-to-access children had been vaccinated.

This study was subject to several limitations. First, combining multiple sources of information could be problematic. In this survey, if vaccination doses were counted twice due to mistakes made in recording of dates, combining sources could have erroneously increased the total reported number of vaccination doses. However, this concern is limited to the few children who were not considered completely vaccinated in any single source independently, but required vaccinations recorded in multiple sources to be combined (2%, 5%, and 9% among children aged one, two, and six years, respectively). Second, even with three sources of data, there may have been incomplete information. For example, fully-vaccinated children who recently moved to CNMI may not have had accurate health or registry records in CNMI. Third, as with any survey, selected children may not have been representative of all children in CNMI. Survey weights based on probability of selection were used to ensure that results from surveyed children were representative of all children. However, some bias may remain due to missed households or differential nonresponse; bias could be further reduced through use of nonresponse-adjusted and poststratified weighting schemes. Nevertheless, interviewers were able to determine household-reported eligibility for 94% of households, and >99% of those with eligible children agreed to participate, minimizing potential bias. Finally, we were not able to evaluate person-days required to conduct follow-up visits, as this information was not documented during the survey.

## Conclusion

Methodological rigor should be maximized to the extent possible in public health surveys. In addition to selecting an appropriate sampling methodology, study designers should carefully consider how to minimize other potential sources of error. Utilizing the most accurate and possibly multiple sources of information, conducting follow-up visits to households, and including difficult-to-access areas may help to reduce survey error and improve validity of results. When any of these strategies is not feasible, limitations should be well-documented and the likely direction and magnitude of bias should be considered and discussed.

While others have discussed the importance of balancing survey quality and cost [13,17], we found little published documenting the effects of the survey "shortcuts" that are presented here. Additional studies to evaluate these issues should be conducted in various settings to document the potential range of their effects and their relative importance in ensuring accurate and representative data on which to base sound public health actions and policy.

## Abbreviations

US Centers for Disease Control and Prevention, CDC; US Commonwealth of the Northern Mariana Islands, CNMI; diphtheria-pertussis-tetanus vaccine, DPT; *Haemophilus influenzae *type b vaccine, Hib; inactivated poliovirus vaccine, IPV; measles-mumps-rubella vaccine, MMR; Office of Management and Budget, OMB.

## Competing interests

The author(s) declare that they have no competing interests.

## Authors' contributions

EL supervised the study, developed the study design, participated in acquisition of data, conducted the statistical analysis, and drafted the manuscript. SS participated in acquisition of data and assisted with the study design and interpretation of the data. KS assisted with the study design, statistical analysis, and interpretation of data. MS and MM participated in acquisition of data. All authors participated in critical revision of the manuscript and approved the final manuscript.

## Pre-publication history

The pre-publication history for this paper can be accessed here:



## References

[B1] (1999). Evaluation of Multiple Indicator Cluster Surveys.

[B2] US Agency for International Development, (2005). Demographic and Health Survey Supervisor's and Editor's Manual. MEASURES DHS Basic Documentation No 5.

[B3] Smith PJ, Battaglia MP, Huggins VJ (2001). Overview of the sampling design and statistical methods used in the National Immunization Survey. Am J Prev Med.

[B4] Botman SL, Moore TF, Moriarty CL, Parsons VL (2000). Design and estimation for the National Health Interview Survey, 1995–2004. National Center for Health Statistics, Centers for Disease Control and Prevention Vital Health Stat.

[B5] Lemeshow S, Tserkovnyi AG, Tulloch JL, Dowd JE, Lwanga SK, Keja J (1985). A computer simulation of the EPI survey strategy. Int J Epidemiol.

[B6] Katz J, Yoon SS, Brendel K, West KP (1997). Sampling designs for xerophthalmia prevalence surveys. Int J Epidemiol.

[B7] Brogan D, Flagg EW, Deming M, Waldman R (1994). Increasing the Accuracy of the Expanded Programme on Immunization's Cluster Survey Design. Ann Epidemiol.

[B8] Turner AG, Magnani RJ, Shuaib M (1996). A Not Quite as Quick but Much Cleaner Alternative to the Expanded Programme on Immunization (EPI) Cluster Survey Design. Int J Epidemiol.

[B9] Milligan P, Njie A, Bennett S (2004). Comparison of two cluster sampling methods for health surveys in developing countries. Int J Epidemiol.

[B10] Lanata CF, Black RE (1991). Lot quality assurance sampling techniques in health surveys in developing countries: advantages and current constraints. Wld Hlth Statist Quart.

[B11] Lemeshow S, Taber S (1991). Lot quality assurance sampling: single- and double-sampling plans. Wld Hlth Statist Quart.

[B12] Robertson SE, Valadez JJ (2006). Global review of health care surveys using lot quality assurance sampling (LQAS), 1984–2004. Soc Sci Med.

[B13] Groves RM (1989). Survey errors and survey costs.

[B14] U.S. Census Bureau (2003). 2000 Census of Population and Housing, Commonwealth of the Northern Mariana Islands Summary File: Technical Documentation.

[B15] Luman ET, Sablan M, Anaya G (2007). Vaccination coverage in the US Commonwealth of the Northern Mariana Islands, 2005. J Public Health Manag Pract.

[B16] (2006). U.S. Office of Management and Budget. Standards and guidelines for statistical surveys. http://www.whitehouse.gov/omb/inforeg/statpolicy.html.

[B17] World Health Organization (2005). Immunization Coverage Cluster Survey – Reference Manual.

